# Optimism for mitigation of climate warming impacts for sea turtles through nest shading and relocation

**DOI:** 10.1038/s41598-018-35821-6

**Published:** 2018-12-04

**Authors:** Nicole Esteban, Jacques-Olivier Laloë, Fionne S. P. L. Kiggen, Selma M. Ubels, Leontine E. Becking, Erik H. Meesters, Jessica Berkel, Graeme C. Hays, Marjolijn J. A. Christianen

**Affiliations:** 1 Department of Biosciences, Swansea University, Singleton Park, Swansea, SA2 8PP UK; 2 Centre for Integrative Ecology, School of Life and Environmental Sciences, Deakin University, Warrnambool, Victoria, 3280 Australia; 3 Marine Animal Ecology Group, Wageningen University, Wageningen, NL-6700 AA The Netherlands; 4 Wageningen Marine Research, Ankerpark, Den Helder, NL-1781 AG The Netherlands; 5 St Eustatius National Parks Foundation, Gallows Bay, St Eustatius, Netherlands; 6 Aquatic Ecology and Water Quality Management Group, Wageningen University, Wageningen, NL-6700 AA The Netherlands; 7 Groningen Institute for Evolutionary Life Sciences, University of Groningen, P.O. Box 11103, 9700 CC Groningen, The Netherlands

## Abstract

Increasing incubation temperatures may threaten the viability of sea turtle populations. We explored opportunities for decreasing incubation temperatures at a Caribbean rookery with extreme female-biased hatchling production. To investigate the effect of artificial shading, temperatures were measured under simple materials (white sheet, white sand, palm leaves). To test natural drivers of incubation temperature, temperatures were measured at average nest depths with shading on two beaches. Results from a pilot experiment suggest the most effective material was palm leaves. Shading decreased temperatures by a mean of 0.60 °C (SE = 0.10 °C, N = 20). Variation between beaches averaged 1.88 °C (SE = 0.13 °C, N = 20). We used long-term rookery data combined with experimental data to estimate the effect on sex ratio: relocation and shading could shift ratios from current ranges (97–100% female) to 60–90% female. A conservation mitigation matrix summarises our evidence that artificial shading and nest relocation are effective, low-cost, low-technology conservation strategies to mitigate impacts of climate warming for sea turtles.

## Introduction

Climate change continues at unprecedented rates with well-documented impacts across terrestrial and marine environments, primarily affecting ecosystem function, species abundance and distributions^1^. Climate change models indicate an increase in temperature, sea level and precipitation^2^. These predictions are highly relevant to oviparous reptiles due to direct ecological threats affecting nest flooding, nesting site availability and suboptimal sex allocation in species with environmental sex determination^3^. Furthermore, population-scale consequences associated with increased temperatures arise in taxa exhibiting temperature-dependent sex determination (TSD), such as reptiles and some species of fish^4^. Hence understanding how climate change will impact sex ratios and population viability in sea turtles, and how these impacts may be mitigated, have been identified as key issues^5^.

Sea turtles exhibit TSD with female hatchlings produced at higher temperatures, males at cooler temperatures and a balanced sex ratio at the pivotal temperature (around 29 °C)^6^. Higher temperatures not only increase female-biased sex ratios but also increase egg mortality, with these effects being consistent across populations and species^7,8^. With the average global temperature predicted to increase by at least 2.6 °C by 2100^9^, warming temperatures threaten sea turtles through effects on hatching sex ratio skews and increased hatchling mortality^10^.

While there has been long-term concern over the status of sea turtles, conservation efforts around the world have led to increases in nesting numbers for a wide range of populations and species^11^. Yet climate warming remains a threat for the viability of this group broadly and so assessing the effects of climate change was identified as a top global research priority for successful future conservation of turtles^5^ and quantification of the effect of warming temperatures is a conservation priority. Recent research efforts have focused on current and predicted hatchling sex ratios^7^ with concerns that high female-biased primary sex ratios are already common at most sea turtle rookeries, exceeding 3:1 in >50% studies^12^. Many sea turtle rookeries are producing female-biased populations (e.g., Brazil^13^, Caribbean^14,15^, Florida^16^, Mediterranean^17^, Australia^8^), or will skew towards near feminisation of hatchling output within 50 years (e.g. Australia^18^).

There are relatively few sea turtle rookeries where cooler sand temperatures produce balanced hatchling sex ratios. Geographic locations at the latitudinal extreme of nesting ranges is an obvious driver of cooler temperatures, for example South Brazil and North Carolina in the Atlantic^10,19^. Natural beach variability and vegetation shading are important drivers for cooler temperatures on a remote Indian Ocean atoll^20^. Other studies have shown that coastal vegetation shading can offset or delay climate change driven female-biased primary sex ratios, e.g., Guadeloupe, Caribbean^21^. However, for sea turtle rookeries without coastal vegetation (e.g. Great Barrier Reef islands in Australia, Ascension Island and some Cape Verdean islands in the Atlantic), the restricted natural variation in beach temperatures provides little or no resilience for a balanced primary sex ratio in future. This has led to the development of management interventions to conserve turtle populations, and  options for decreasing incubation temperatures include artificial shading^22^, watering and relocation to greater sand depths^23^. While the general impact of shading, increased depth and watering to cool nests is well known, it is not known if the magnitude of these impacts is invariant across sites and hence whether they will provide the same impact as a management intervention across the world. Hence further trials of methods to artificially cool nests are needed.

The present study explores opportunities for decreasing incubation temperatures at a rookery with extreme female-biased hatchling production in St Eustatius, North East Caribbean. Recent incubation temperature studies suggested that three species nesting at this rookery (leatherback turtle, *Dermochelys coriacea*, hawksbill turtle, *Eretmochelys imbricata* and green turtle, *Chelonia mydas*) have had female-biased hatchling production for at least a century with <36% males produced every year^14^. In this study, our aim was to examine the thermal effect of different shading techniques and consequent hatchling sex ratio. (1) We investigate the most efficient low-technology, low-cost shading technique. (2) Informed by the results of shading experimentation, we examine the effect of environmental variables (beach, shade, depth) on temperature and hatchling sex ratio. (3) Using the results from the effect of different environmental variables, predict potential benefits of conservation actions and likely consequence for current and future primary sex ratios at a rookery with extreme female bias.

## Results

### Assessment of shading techniques

Data were successfully retrieved from the six loggers on Zeelandia Beach. All loggers collected data for a period of 69 hours.

**Figure 1:**
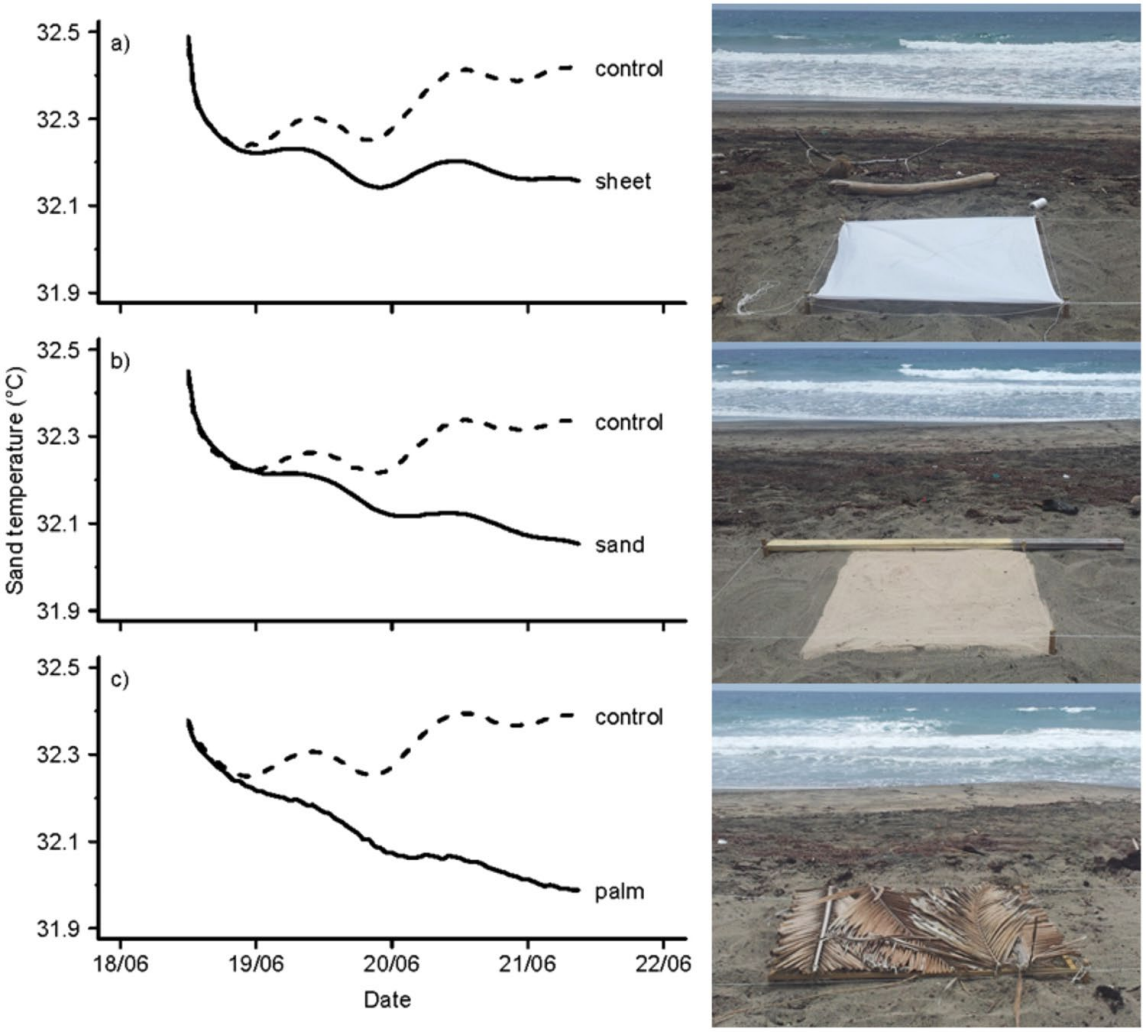
Three different shading techniques were used to cool sand temperatures at mean hawksbill and green turtle nest depth (50 cm). The maximum difference between sand temperatures recorded under the white cotton sheet (**a**) and corresponding control temperatures was 0.26 °C. The maximum difference between sand temperatures recorded under the white sand (**b**) and control temperatures was 0.28 °C. The maximum difference between sand temperatures recorded under the palm leaves (**c**) and control temperatures was 0.40 °C. This pilot experiment ran from 18 June 2012 until 21 June 2012 and provides a conservative estimate as the temperature differences between controls and shading treatments were expected to increase further after these 3 days.

All three treatments were effective at cooling sand temperatures (Fig. 1). At the end of the experiment (i.e. after 69 hours) sand temperature recorded under the white cotton sheet was 0.26 °C lower than the corresponding control temperature. Temperature recorded under the white sand was 0.28 °C lower than its corresponding control temperature, and temperature under the palm leaves was 0.40 °C lower than its corresponding control temperature.

To quantify the effect of shading on temperature we calculated the difference between sand temperatures recorded in a treatment plot (i.e. shaded with white cotton sheet, white sand or palm leaves) and sand temperatures recorded in the associated control plot. To reduce the effect of temporal auto-correlation while retaining enough points for analysis we used 5-hour means in the analysis. An analysis of covariance confirms that treatment had an effect on sand temperature (F5,36 = 367.3, p < 0.05, 95% Confidence Interval (CI) = −0.0355, −0.0308). While all three treatments successfully lowered sand temperatures, palm leaves proved to be the most effective treatment, reducing sand temperatures by approximately 0.3 °C during the first 50 hours of the experiment (F1,12 = 498.6, p < 0.05, CI = −0.0363, −0.0299). Using white sand as a treatment reduced sand temperatures by approximately 0.2 °C during the first 50 hours of the experiment (F1,12 = 476.7, p < 0.05, CI = −0.0268, −0.0219). Using the white cotton sheet also reduced sand temperatures by approximately 0.2 °C during the first 50 hours of the experiment (F1,12 = 918.6, p < 0.05, CI = −0.0235, −0.0203). At the end of 3 days, the difference between temperatures under treatment plots and temperatures under the corresponding control plots continued to show a linear and decreasing trend. Realistically, these differences should gradually reach horizontal asymptotes. Unfortunately our experiment did not run long enough to reveal this. Regardless, the results from this short pilot experiment informed with confidence which treatment was most effective at cooling sand temperatures (see Fig. 1).

### Effect of depth, shade and beach on sand temperature

**Figure 2:**
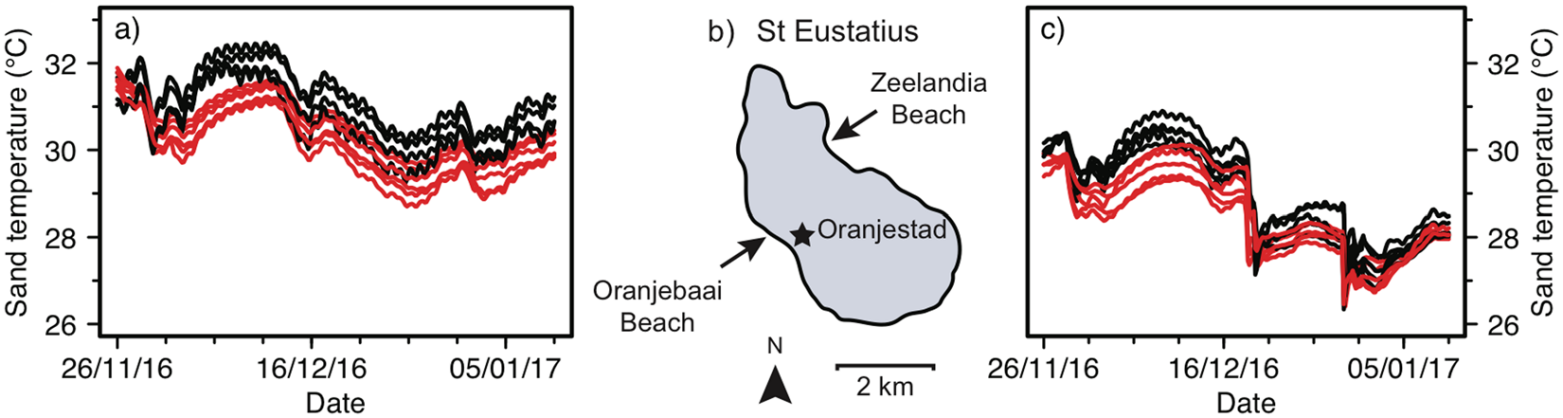
Sand temperatures recorded on St Eustatius between 26 November 2016 and 10 January 2017. A total of 20 temperature loggers recorded sand temperatures on (**a**) Oranjebaai Beach (n = 4 for control, n = 6 treatment) and (**c**) Zeelandia Beach (n = 5 for control, n = 5 treatment) in control plots (black lines) and plots shaded by palm leaves (red lines) at mean turtle nest depth (50 and 63 cm).

Four loggers were lost due to beach erosion. The remaining 20 loggers were successfully retrieved and included in the analysis. Data from 26 November 2016 until 10 January 2017 were used for the analyses, covering a 46-day period (Fig. 2).

To study the relationship between sand temperature, depth (i.e. 50 vs 63 cm), treatment (i.e. control vs shaded) and beach (i.e. Oranjebaai Beach vs Zeelandia Beach) mean sand temperatures were calculated for each logger. We performed a linear mixed model analysis to understand the relationship between sand temperature, depth, treatment and beach. Depth, treatment and beach were entered as fixed effects. Site and plot were entered as random effects. Normality of the residuals was checked through visual inspection of the residual plots. Likelihood Ratio Tests were used to obtain P-values. We tested the significance of the three-way and all two-way interactions by comparing a model with no interactions to a model with one interaction of interest.

**Figure 3:**
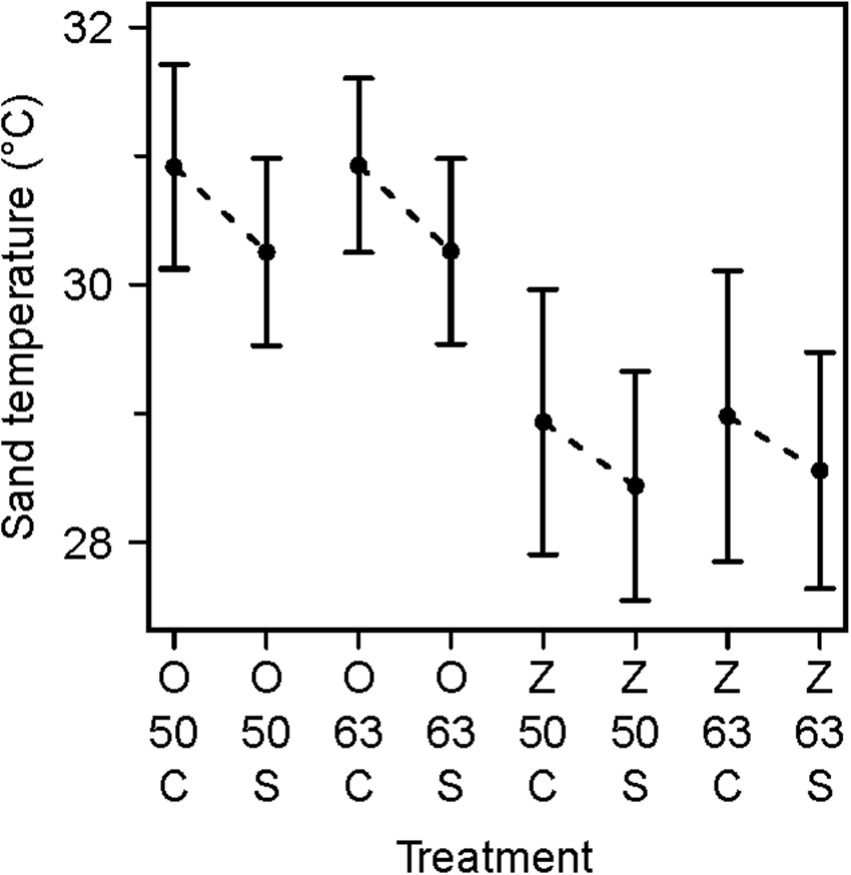
Beach and treatment had an effect on sand temperature. Mean sand temperatures are given for each group of loggers in a treatment plot (i.e. shaded) and associated control plot. The intervals define the standard deviation. O: Oranjebaai Beach; Z: Zeelandia Beach; 50: 50 cm; 63: 63 cm depths; C: Control; S: Shaded. The dashed lines highlight the difference between a control plot and the corresponding shaded plot situated on the same beach and at the same depth.

The main effect of beach on sand temperature was clearly visible in our data, with sand temperatures on Oranjebaai Beach being approximately 2.0 °C higher than sand temperatures on Zeelandia Beach (Figs 2, 3). Similarly, the effect of treatment (control vs shaded) is clear, with shaded plots being approximately 0.6 °C cooler than control plots (Figs 2, 3). In contrast, depth visibly shows no effect on sand temperatures (Fig. 3). The effect of shading increased by a factor of 2 (from approximately 0.3 °C to 0.6 °C) and results from the earlier shorter experiment are a conservative estimate. None of the interactions were significant (χ^2^ = 5.27, df = 4, p = 0.26) so we used a model with no interactions. Our model confirms that beach had a significant effect on sand temperature (χ^2^ = 21.34, df = 1, p < 0.05, CI = −2.1801, −1.5885). Treatment also had a significant effect on sand temperatures (χ^2^ = 14.46, df = 1, p < 0.05, CI = −0.8433, −0.3637) while depth did not (χ^2^ = 1.66, df = 1, p = 0.20).

We constructed a conservation mitigation matrix relating temperature changes between study beaches and treatments (Table 1). The biggest temperature difference was found between control plots on Oranjebaai Beach and shaded plots on Zeelandia Beach (±2.487 °C).

**Table 1:** Sand temperature variations (between shading treatment and beaches) provide guidelines for potential conservation actions to decrease sea turtle nest incubation temperature.

	New location				
	X	O C	O S	Z C	Z S
**Source location**	**O C**	X	−0.60 °C (±0.10 °C)	−1.88 °C (±0.13 °C)	**−2.48 °C** (±0.23 °C)
	**O S**	0.60 °C (±0.10 °C)	X	−1.28 °C (±0.23 °C)	−1.88 °C (±0.13 °C)
	**Z C**	1.88 °C (±0.13 °C)	1.28 °C (±0.23 °C)	X	−0.60 °C (±0.10 °C)
	**Z S**	2.48 °C (±0.23 °C)	1.88 °C (±0.13 °C)	0.60 °C (±0.10 °C)	X

Sand temperatures at Zeelandia Beach vary from 31.9–33.3 °C in peak nesting season^14^. Values represent the difference between a source location (rows) and a new location (columns). Column and row headings represent beach location (O = Oranjebaai Beach; Z = Zeelandia Beach) and treatment (S = shading by palm leaves; C = control). Values in brackets are the standard errors (n = 20).

### Primary sex ratios

Mean monthly sand temperatures on Zeelandia Beach are reported to vary from 29.1–29.6 °C in January–March to 31.9–33.3 °C in June–August^14^. Using the equation describing the relationship between primary sex ratio and incubation temperature^7^, it is possible to estimate that the primary sex ratios are currently approximately 50–56% female-biased in January–March and 97–100% female-biased in June–August.

Lowering sand temperatures through the implementation of mitigation strategies such as relocating eggs from Oranjebaai Beach to Zeelandia Beach and shading turtle nests would have an effect on sex ratios (Table 2). For example, a change of 1.0 °C would result in primary sex ratios being approximately 21–34% female-biased during January–March and 91–98% female-biased in June–August. A decrease of 2.5 °C would result in primary sex ratios being approximately 4–7% female-biased during January–March and 60–90% female-biased in June–August.

**Table 2:** Current and predicted primary sex ratios indicate the potential benefits of implementing conservation mitigation strategies.

	Temperature change (°C)			
	Current	−1	−2	−2.5
January–March	50–56%	21–34%	7–12%	4–7%
June–August	97–100%	91–98%	74–95%	60–90%
	**2090**	**−1 °C**	**−2 °C**	**−2.5 °C**
January–March	98–99%	95–97%	83–90%	71–83%
June–August	100%	100%	99–100%	99–100%

Current values are based on incubation temperatures recorded on St Eustatius in 2011-2012^14^ calculated using the relationship between incubation temperature and primary sex ratio^7^. 2090 values are based on incubation temperatures projected for the year 2090^14^. Two seasonal periods are provided: June–August are peak nesting months; occasional nesting by hawksbills and leatherbacks occurs during January–March (JB, NE, unpublished data). All values are given as female percentages (e.g. 60% indicates 60% females and 40% males in a clutch).

It is projected that mean incubation temperatures on St Eustatius will increase by approximately 1.1 °C by the year 2030, by 2.0 °C by 2060 and by 3.2 °C by 2090^14^. Without mitigation strategies, this will result in primary sex ratios being close to 100% female by the end of the century. Nest relocation or artificial shading would only have a measurable effect on primary sex ratios during January–March (Table 2).

## Discussion

Our results demonstrate that simple, low cost, low technology artificial shading can have a significant impact on sea turtle incubation conditions. All shading strategies reduced temperature and the most effective treatment was palm leaves, supporting results of previous studies^24–26^. This contrasts to a previous shading experiment (using solar wave fabric) that had variable results^27^. Furthermore, we report that relocation to a cooler windward beach can reduce the temperature of the eggs by a factor of four. We calculated the effects of these simple conservation actions, which would result in a positive shift from a female-skewed to a balanced primary sex ratio in the current conditions. The application of our results to mitigation strategies provides grounds for cautious optimism that simple and low-cost conservation actions can be effective at rookeries with high incubation temperatures and female-skewed primary sex ratios.

The effects in temperature decrease due to artificial shading at our study site are comparable to the magnitude of effects of shading by coastal vegetation and forest border on naturally shaded nesting beaches^20,21^. This suggests that artificial shading can be a substitute for natural vegetation shading at sea turtle rookeries in arid environments (e.g. Great Barrier Reef islands). Depth does not have such a clear effect as shading and differs due to a delayed time-lag response during periods of cooling and warming as well as a reduced pattern of diel variation at depth^14,20^. Relocation of nests to different depths that reflect depths of natural nests at the same beach may not have the anticipated results because of complicated interactions between depth and the environment. However, our experiments were not designed to look at the full extent of depth impacts, since we only varied the range of depths fairly minimally (50–63 cm) corresponding to the mean natural depth of sea turtle nests on the island, while the difference between the minimum and maximum depth of incubating eggs on the study beach is likely to have a much greater effect. Although relocation practices can negatively affect egg and hatchling survivorship^28^, examples of successful relocation conservation programmes exist worldwide^29,30^. Our results further demonstrate that shading in combination with relocation to a different cooler beach can have much greater benefits for a balanced primary sex ratio.

While results from experiments studying the effect of incubation temperature on emergence success have been inconclusive for St Eustatius^14^, it is possible to use published relationships to estimate the impact mitigation strategies would have on emergence successes. For example, at an incubation temperature of 32.5 °C, emergence success is likely to be <50%^7^. Lowering temperatures by 2 °C through mitigation strategies (e.g. relocation) could increase the emergence success to >80%. A further 0.5 °C reduction in incubation temperature (e.g. shading) would bring emergence successes close to 85%. This serves to illustrate the impact mitigation strategies can have on both primary sex ratios and emergence successes of sea turtles.

The variation in thermal ecology of two nesting beaches (<1 km distance) at our study site was surprising as the sand on both beaches is of similar (volcanic) origin and within-beach variation was not significant in a previous study^14^. The strong northeastern trade winds^31^ are likely to provide a cooling effect for Zeelandia Beach. At this cooler beach, temperatures are low enough to produce a balanced primary sex ratio during off-peak nesting season in current conditions. There is evidence that turtles select particular micro-habitats for nesting. For example, hawksbill turtles nest under vegetation^32^, loggerheads *Caretta caretta* nest on mangrove islands^33^ and painted turtles *Chrysemys picta* seek shaded nest locations at warmer sites^34^. Protection of rookeries that appear minor in terms of numbers may be essential for the health of the larger regional population^19^. Similarly, beaches with a range of shade cover should be identified to allow plasticity in nest-site choice^21^. It is important to take a regional approach to assess temperature at all rookeries and identify high priority cool beaches for protection.

When projecting into the future, warming air temperatures are predicted to cause the mean incubation temperatures at our study site to reach 32.1 °C by 2030 and 34.2 °C by 2090^14^, above the upper thermal limit of 33 °C for successful incubation^35^. Alongside warming air temperatures, global sea level rise will inundate beaches and limit available nesting habitat^36^. In the meantime, these temperatures will exacerbate existing female bias: female green turtle sex ratios have been >95% since 2009 and female sex ratios of leatherbacks and hawksbills are projected to reach >95% by the years 2028 and 2045 respectively^14^. A key issue remains whether phenological shifts in the seasonal timing of nesting will help mitigate predicted impacts of climate warming. In the absence of phenological shifts in nesting or conservation actions, it is possible that this rookery will no longer support viable nesting turtle populations. However, we urge caution with extending these conclusions to other areas since our results are from a rookery with extreme sand temperatures. Relocating and shading of nests at other rookeries is likely to produce different results than those reported in this study, so we emphasize that our results should be perceived as guidelines only. A vulnerability assessment framework^37^, microclimate model^38^ or sensitivity map^39^ may assist with gaining a thorough understanding of natural variability of beach temperatures before planning conservation strategies such as relocation or artificial shading. A further issue for consideration is the practicality of management interventions, i.e. how to scale up cooling treatments of a few days for a handful of sites up to 100 s or 1000 s of nests over several months.

With incubation temperatures projected to rise by up to 4 °C over the next century at this study site^14^, these mitigation strategies may prove essential to guarantee that future operational sex ratios and emergence successes are viable. However, the maximum temperature decrease achievable by relocation and artificial shading is less than this predicted temperature increase, so will only be sufficient to maintain a balanced primary sex ratio in the cooler off-peak nesting seasons. Our model is limited by the use of estimated values for primary sex ratio and emergence success. As shown for other rookeries, it is possible that male embryo survival and different male and female breeding periodicities will mitigate the highly female-skewed primary sex ratio^7,12^. In light of new research that demonstrates that higher temperatures increase the natural growth rate of turtle populations (as more females are produced) but that long-term population survival is threatened (once incubation temperatures near lethal levels), an improved understanding of emergence success may be more of a priority than sex ratios^40^. Finally, if sex ratios of a small and endangered population are significantly skewed, there may be consideration of a captive breeding programme (e.g. a hatchery) to release additional individuals into the wild to support small or declining populations^41^ or an artificial change in the conditions at the egg-laying site^42^. The sudden drops in temperature we attributed to rainfall have also been noted elsewhere on nesting beaches^43^. This impact of rain on nest temperatures highlights how the impacts of changing rainfall patterns also need to be considered when assessing the impact of climate change on sea turtle hatchling sex ratios.

Effective transboundary conservation of migratory species is a difficult challenge faced by many conservation bodies^44^. Management of nesting beaches is a widespread practice for endangered turtle populations^45^. Although results from our study contribute to a greater understanding of conservation strategies to decrease incubation temperature, there is a consensus that greater understanding of risks and effectiveness associated with conservation programmes is required^5^. Several regional sea turtle conservation networks exist, e.g., Wider Caribbean Sea Turtle Network (WIDECAST; www.widecast.org) and the Indian Ocean–Southeast Asian Memorandum of Understanding for the Conservation and Management of marine turtles and their habitats (www.ioseaturtles.org). These networks may serve as starting points to identify high priority cool beaches for protection and for discussions about a standardised framework for beach conservation actions at rookeries with extreme female-biased primary sex ratios and that lack natural coastal vegetation to enhance natural resilience in the face of global climate change^46^.

## Methods

### Study site

The 21 km^2^ island of St Eustatius is located in the Lesser Antilles in the north-eastern Caribbean. Almost all clutches are laid by leatherback, hawksbill and green turtles on two beaches: Oranjebaai Beach (sheltered, western coast; 17.483°N, 62.988°W) and Zeelandia Beach (exposed, eastern coast; 17.507°N, 62.980°W), the latter being the most important nesting beach^46^. The typical nesting season is March until June (leatherback), June until November (hawksbill) and July until October (green) (JB, NE, STENAPA unpublished data). The study was conducted within the Statia National Marine Park programme and complied with all relevant local and national legislation.

### Temperature loggers

Tinytag Plus 2 loggers (Tinytag Plus 2 model TGP-4017, Gemini Data Loggers, UK) were used to record sand temperature at depths representative of nests for leatherback, hawksbill and green turtles nesting on Oranjebaai Beach and Zeelandia Beach during 2012-2013 and 2016-2017. Temperature measurements were recorded every hour. The loggers were originally calibrated to United Kingdom Accreditation Service (UKAS) standards and are accurate to <0.5 °C (www.tinytag.info, last accessed on 28 March 2018). To minimize impact on natural conditions during burial of loggers, care was taken to excavate a sand core and then replace it back on top of the logger. This was achieved by hammering a 10 cm diameter PVC pipe to the desired depth of the logger, creating a vacuum and then removing the pipe full of sand. The depth of the hole was verified with a semi-rigid tape measure, then the logger was dropped into the hole and the sand was emptied out of the pipe on top of the logger. A string was connected to the logger to facilitate relocation of the loggers. GPS positions of the loggers were recorded. After burial, one day was allowed for the sand temperatures to return to original state after disturbance. To ensure all loggers were exposed to the same degree, the surface of the experimental area was cleared from seaweed patches, other organic material and debris.

### Assessment of shading techniques

An artificial shading experiment to examine the effects of low-cost and available shade materials on sand temperatures was conducted over a 72 hour period from 18–22 June 2012 on the principal turtle nesting beach, Zeelandia Beach. Loggers were deployed at mean nest depth of 50 cm (hawksbill and green turtles; JB, NE, STENAPA unpublished data). Three shading materials were used: white cotton sheet, white (imported) sand and (local) palm leaves (*Cocos nucifera*) (Fig. 1). An area of 1.5 × 10 m was cleared (i.e. organic material and debris were removed) and the sand surface was raked flat. Six loggers were buried in a row parallel to the waterline at 1.5 m intervals and 50 cm depth. After burying loggers, artificial shading methods (1 m² surface area) were immediately placed on the sand surface so that each logger was centrally located within the experimental plot.

### Effect of depth, shade and beach on sand temperature

Loggers were buried at mean nest depths of 50 cm (hawksbill and green turtles) and 63 cm (leatherback turtles). Mean depths were calculated from records as the midpoint between the top and bottom of clutches of eggs excavated between 2005–2015 (JB, NE, STENAPA unpublished data). To examine the key drivers of sand temperature we measured the impact of shading and nest depth at two beaches: Oranjebaai Beach (Southwest coast) and Zeelandia Beach (Northeast coast). At each beach three different plots were selected as replicates. In total, 24 loggers were buried: 12 were buried at each site (beach); at each site 4 were buried in each plot (replicates); within each plot 2 were buried for each treatment (control vs shaded); within each treatment 1 logger was buried at each depth (50 or 63 cm). The experiments were conducted from 23/09/2016–22/11/2016 and from 25/11/2016 until 09/01/2017. Initially the Oranjebaai Beach experiment was at the north end. A heavy storm on 17/11/2016 eroded the beach, several loggers were lost and loggers were relocated (using the same treatments) to the south end of Oranjebaai Beach. Shaded areas were created using palm leaves attached to 1 m² wooden frames with biodegradable cotton string and placed over the buried loggers. The wooden frames were made using branches from small native trees. The palm leaves were collected from the ground next to palm trees around St Eustatius.

Data were downloaded from temperature loggers using TinyTag Explorer 4.7. Prior to the analyses, data from before logger deployments, during relocation periods, and from after retrievals were discarded. All datasets were reviewed and checked for anomalies. We used R^47^ and package lme4^48^ for analyses.

### Primary sex ratios

The relationship between incubation temperature and primary sex ratio^7^ was used to estimate the potential that mitigation strategies have to affect sex ratios at this field site. We used this model to calculate the expected change in primary sex ratios with cooling regimes of 1 °C, 2 °C and 2.5 °C.
